# Comparison of Sample Preparation Methods for Shotgun Proteomic Studies in Aquaculture Species

**DOI:** 10.3390/proteomes9040046

**Published:** 2021-11-16

**Authors:** Mário Jorge Araújo, Maria Lígia Sousa, Aldo Barreiro Felpeto, Maria V. Turkina, Elza Fonseca, José Carlos Martins, Vítor Vasconcelos, Alexandre Campos

**Affiliations:** 1 CIIMAR-UP-Interdisciplinary Centre of Marine and Environmental Research, University of Porto, Terminal de Cruzeiros do Porto de Leixões, Av. General Norton de Matos, s/n, 4450-208 Porto, Portugal; mario.araujo@ciimar.up.pt (M.J.A.); msousa@ciimar.up.pt (M.L.S.); aldo.barreiro@gmail.com (A.B.F.); fonseca.ess@gmail.com (E.F.); jmartins@ciimar.up.pt (J.C.M.); vmvascon@fc.up.pt (V.V.); 2 Department of Biomedical and Clinical Sciences, Faculty of Medicine and Clinical Sciences, Linköping University, 581 83 Linköping, Sweden; maria.turkina@liu.se; 3 Biology Department, Faculty of Sciences, University of Porto, Rua do Campo Alegre, s/n, 4169-007 Porto, Portugal

**Keywords:** aquaculture, *Mytilus galloprovincialis*, *Scophthalmus maximus*, protein profiling, functional analysis, FASP, SP3, S-Trap

## Abstract

Proteomics has been recently introduced in aquaculture research, and more methodological studies are needed to improve the quality of proteomics studies. Therefore, this work aims to compare three sample preparation methods for shotgun LC–MS/MS proteomics using tissues of two aquaculture species: liver of turbot *Scophthalmus maximus* and hepatopancreas of Mediterranean mussel *Mytilus galloprovincialis*. We compared the three most common sample preparation workflows for shotgun analysis: filter-aided sample preparation (FASP), suspension-trapping (S-Trap), and solid-phase-enhanced sample preparations (SP3). FASP showed the highest number of protein identifications for turbot samples, and S-Trap outperformed other methods for mussel samples. Subsequent functional analysis revealed a large number of Gene Ontology (GO) terms in turbot liver proteins (nearly 300 GO terms), while fewer GOs were found in mussel proteins (nearly 150 GO terms for FASP and S-Trap and 107 for SP3). This result may reflect the poor annotation of the genomic information in this specific group of animals. FASP was confirmed as the most consistent method for shotgun proteomic studies; however, the use of the other two methods might be important in specific experimental conditions (e.g., when samples have a very low amount of protein).

## 1. Introduction

Large-scale proteomics has been providing new information for fundamental biology [1,2], and in several related sciences such as toxicology [3,4], animal physiology and pathology [5], food safety, and aquaculture [6,7,8]. It has been providing insights concerning the mechanisms underlying several diseases [9,10]. Proteomics allows a better understanding of molecular pathways and the metabolism of organisms, and it has also been contributing to biomarker research [11,12]. In the field of aquaculture, proteomics is still considered a recent research topic and, therefore, some work is still needed at the methodological and technical level, to improve proteome analysis [4].

Current proteomics methods rely mostly on liquid chromatography (LC) and mass spectrometry (MS). Mass spectrometers have become highly powerful and sensitive instruments allowing the analysis of very complex samples and high-throughput proteome analyses [13]. On the other hand, conventional gel electrophoresis techniques have proven useful in current proteomics research by improving the fractionation of highly complex samples [14,15,16,17]. Moreover, the existence of new sample preparation techniques such as filter-aided sample preparation (FASP) is also making proteomic studies more reliable and sensitive, in addition to contributing to the identification of specific pathways or protein interaction networks and the determination stoichiometry of protein complexes [13].

The access to full genome sequences has been the key to the use of MS techniques in proteomics research, allowing the identification of proteins and their expression from one cell, individual, or community, out of a complex mixture of peptides digested with a specific protease (e.g., trypsin), followed by mass spectrometry (MS/MS approach) and searching using online databases, such as UNIPROT (https://www.uniprot.org) or NCBI (https://www.ncbi.nlm.nih.gov/sites/batchentrez).

Sample preparation in modern high-throughput proteomic studies (such as shotgun analysis) differs considerably from previous gel-based approaches, due to the need to combine protein extraction and digestion prior to MS analysis. The three most common sample preparation methods are FASP, suspension-trapping (S-Trap), and single-pot, solid-phase-enhanced (SP3). Prefractionation steps, such as size exclusion chromatography, hydrophobic interaction chromatography, reverse-phase chromatography, weak anion exchange and strong anion exchange chromatography, liquid- and gel-phase isoelectric focusing chromatography, or 1D-PAGE [15,18], can be employed during sample preparation. Such prefractionation steps aim to improve the identification and quantification of minor or under-represented proteins in a complex sample.

Other steps, aiming to minimize the effects of the use of detergents, are usually employed during sample preparation. Indeed, detergents such as sodium dodecyl sulfate (SDS) are needed to ensure a good solubilization and extraction of proteins from the biological materials. However, most detergents are not compatible with MS analysis because they are easily ionized, hiding the general peptide signals from the sample in mass spectrometers [19]. Detergents can also inhibit protease activity and the enzymatic digestion of the proteins into peptides. Salts (including sodium chloride) are also detrimental to MS analysis and, therefore, most sample preparation protocols include washing/sample desalting steps.

The FASP method, compatible with a broad variety of salts and detergents, enables the exchange of the detergent-solubilized proteins to a chaotropic agent (urea) using a filtering device for the digestion into peptides. The eluted peptides can then be desalted using C-18 columns, prior to MS analysis. The S-Trap technique allows the formation of a protein suspension from SDS using a combination of phosphoric acid and methanol. The suspension is then trapped on porous material, and residual SDS is washed away. Further in-filter enzymatic digestion and final peptide elution steps are similar to the FASP technique [20]. The SP3 method employs a hydrophilic interaction mechanism for protein binding to paramagnetic beads to perform buffer exchange, removal of detergents, and protein digestion and concentration [21]. While the FASP method has been successfully used for a wide range of biological samples (including mammals and invertebrates) [22] and modified depending on the purposes of the studies, S-Trap and SP3 methods are relatively less used and characterized [23,24,25].

The liver of vertebrates and hepatopancreas or the digestive gland of invertebrates is of high interest in animal health research, since an important part of the function of these organs is related to energy metabolism, lipid and nutrient storage, immune response, and detoxification in the animal [8,12,26]. However, the high lipid content present in liver and hepatopancreas tissues has posed limitations to proteomics investigations, making it often necessary to modify and develop specific sample preparation protocols to enable an adequate analysis of the proteome from these organs [27,28,29,30,31,32].

Hence, the main aim of this work was to compare the performance of three sample preparation methods commonly used in shotgun proteomics investigations on liver and hepatopancreas samples from two important aquaculture species, turbot *Scophthalmus maximus* and Mediterranean mussel *Mytilus galloprovincialis*, as well as to understand their differences in terms of protein identification, proteome coverage, functional analysis, and pathway identification.

## 2. Materials and Methods

### 2.1. Sample Preparation

Juveniles of turbot *S. maximus* (approximately 125 g total weight) were provided by a commercial fish farm (Acuinova, Mira, Portugal). Liver samples (three replicates, each replicate combining tissues from three organisms) were collected from turbots after organisms were anesthetized with 2-phenoxyethanol (Acros Organics, Geel, Belgium) and euthanized by decapitation. Wild adults of Mediterranean mussel *M. galloprovincialis* (approximately 35 g total weight) were captured at Portuguese Atlantic Coast (41.047755, −8.654146) in May 2020 during the low tide. After arriving to the laboratory, mussels were dissected, and samples of hepatopancreas were collected and pooled (three replicates, each replicate with tissues from three organisms). Samples were frozen after collection and kept at −80 °C until further processing.

### 2.2. Protein Extraction

The samples (liver and hepatopancreas) were defrosted in a box with ice and incubated in SDT buffer (0.5 g fresh weight/mL). SDT buffer was composed of 2% SDS, 0.1 M Tris/HCl pH 7.6, and 0.1 M dithiothreitol with 1:100 protease inhibitor (Halt PI Cocktail CAT #78429, Thermo Scientific, Waltham, MA, USA) at room temperature (RT, 21 °C) for 20 min. After sonication, involving six cycles of 3 s each with a potency of 60% 10 W, VC 50 (Sonics & Materials Inc., Danbury, CT, USA), samples were incubated in the dark at RT for 2 h and then heated for 3 min at 95 °C. Then, samples were centrifuged at 16,000× *g* at 21 °C followed by incubation at RT for 20 min. The supernatant was collected in new Eppendorf tubes, and total protein concentration was estimated by absorbance measurement at 280 nm (A280 application, DeNovix, Ds-11 FX Spectrophotometer: Wilmington, DE, USA). Samples were stored at −80 °C until further treatment.

### 2.3. Sample Preparation for LC-MS Analysis

Protein samples were processed by one of the following methods (three replicates for each method and each tissue): 1—filter-aided sample preparation (FASP), 2—single-pot, solid-phase-enhanced sample preparation (SP3), or 3—suspension-trapping (S-Trap), as described below.

#### 2.3.1. Filter-Aided Sample Preparation (FASP)

The FASP method, described by Wiśniewski et al. (2009) [19], was carried out with modifications. Protein samples (40 µg) were initially diluted in 200 µL of UA buffer (8 M urea, in 0.1 M Tris/HCl, pH 8.5). Samples were then transferred to filter units (30 kDa MWCO, MRCF0R030, Merck, Tullagreen, Ireland) previously washed in water and UA buffer, before undergoing 10 s centrifugation. Then, 100 µL of 0.05 M iodoacetamide was added to the filter units, mixed for 1 min (RT, Thermomixer, Eppendorf, Hamburg, Germany), and incubated without mixing for 20 min. Then, filter units were centrifuged at 14,000× *g* for 10 min. Two successive washings were performed with 100 µL UA buffer, followed by centrifugation for 15 min and 100 µL of 0.05 M ammonium bicarbonate. Digestion of peptides was performed by adding trypsin proteomics grade (CAT #3708985001, Roche, Mannheim, Germany) at a 1:100 enzyme-to-protein ratio in 0.05 M ammonium bicarbonate. For digesting peptides, samples with trypsin were initially mixed for 1 min in Thermomixer (RT, Hamburg, Germany) and then incubated in a wet chamber at 37 °C for 16 h overnight. The obtained peptides were eluted to new tubes by centrifugation (14,000× *g*, 10 min). Further elution was performed with 0.5 M NaCl followed by centrifugation, and the eluates were pooled. Peptide concentrations were measured at 280 nm and acidified with 5 µL of formic acid (0.1% *v/v*). Peptide desalting was performed with C18 columns (C18 UptiTip™ CAT# BI5280, Glygen, Interchim Innovations, Montluçon, France). Conditioning of columns was performed according to the manufacturer’s protocol, i.e., using acetonitrile (60% *v/v*) and formic acid (0.1%, *v/v*). Samples were then added to the columns and washed with formic acid (0.1%, *v/v*). Elution of peptides was finally performed with acetonitrile (60% *v/v*) and formic acid (0.1%, *v/v*) into new tubes. The peptide concentration was measured at 280 nm. The samples were then dried with a vacuum concentrator (CentriVap, Labconco, Kansas City, MO, USA) and stored at −20 °C until further processing.

#### 2.3.2. Single-Pot, Solid-Phase-Enhanced Sample Preparation (SP3)

Prior to protein digestion, samples (approximately 100 µg of protein) were reduced by the addition of 45 µL of SDT buffer. Then, samples were alkylated with 10 µL of 0.1 M iodoacetamide in 8 M urea, 0.1 M Tris/HCl pH 8.5 for 30 min in the dark at RT. Protein digestion was based on the method of Hughes et al. (2019) [21], SP3 for proteomics experiments, with some modifications. New magnetized beads (Sera Mag SP3 beads, CAT#45152105050250 and CAT#65152105050250, GE Healthcare, Little Chalfont, UK) were initially washed and reconstituted in water (HPLC grade, VWR, Lutterworth, UK) at a final concentration of 10 µg/µL (from an initial stock concentration of 50 mg/mL), using a magnetic rack (MagnaRack, Invitrogen, Carlsbad, CA, USA) for 1 min. A 10:1 *v/v* ratio (beads to proteins) was reached by adding 100 µL of the bead suspension to each tube followed by mixing with aspiration movements with a micropipette. Then, 160 µL of absolute ethanol (molecular biology grade, Fisher) was added to achieve 50% *v/v*, also followed by pipette mixing. Incubation was performed in a Thermomixer with shaking for 10 min at RT. Then, sample tubes were placed in the magnetic rack and supernatant was removed. Beads were gently reconstituted in 180 µL of 80% ethanol. Tubes were placed again in the magnetic rack, and the supernatant was discarded. This cleaning step was performed three times. Extraction was then performed gently by adding 100 µL of ammonium bicarbonate (0.1 M pH 8) containing 0.04 µg/µL Trypsin/rLys-C Mix (MS grade, Promega, Madison, WI, USA) to each tube at a 1:50 ratio (enzyme to protein), followed by an incubation period of 14 h at 37 °C with mixing (0.1 g). Then, 1.3 mL of 100% acetonitrile (HPLC grade, Chem-Lab NV, Zedelgem, Belgium) was added and incubated for 18 min. Beads were spun down, and tubes were placed in the magnetic rack. The supernatant was discarded, and beads were washed twice with 180 µL of 100% acetonitrile. After removing the supernatant, peptides were separated from beads by adding 100 µL of ammonium bicarbonate (0.05 M), followed by incubation at RT for 5 min with shaking (Thermomixer). After bead spin and concentrating them with the magnet, the supernatant was recovered and transferred into new tubes. Peptide concentration was estimated at 280 nm. Peptides were acidified by adding 20 µL of formic acid 5% *v/v* (Optima LC/MS grade, Fisher Chemical, Geel, Belgium) to the samples and then concentrated using the CentriVap evaporator centrifuge. Dried samples were then stored at −20 °C until further processing.

#### 2.3.3. S-Trap Sample Preparation

The S-Trap method was performed following the instructions provided by the S-Trap spin column manufacturer (www.protifi.com (accessed on 2 June 2020)). First, protein samples (100 µg) were incubated with 50 µL of SDS 2% (*w/w*), 0.05 M Tris/HCl pH 7.55. Alkylation was then performed with iodoacetamide at a final concentration of 0.014 M, followed by incubation (30 min) in the dark. Then, 5 µL of phosphoric acid (12%) was added followed by S-Trap binding buffer (90% methanol, 0.1 Tris/HCl pH 7.1). The samples were transferred to the S-Trap spin column and centrifuged for 30 s at 4000× *g*. S-Trap columns were washed three times with 400 µL of S-Trap binding buffer. Protein digestion was performed on the S-Trap spin columns with Sequencing Grade Modified Trypsin (Promega, CAT# PROMV5111, Madison, WI, USA) at a 1:50 ratio (enzyme to protein) in 0.05 M ammonium bicarbonate. The digestion was performed in a wet chamber at 37 °C and overnight. Peptides were eluted with 80 µL of 0.05 M ammonium bicarbonate followed by 0.2% formic acid and centrifuged at 1000× *g*. Additional elution was performed with 80 µL of acetonitrile (50%) with 0.2% formic acid followed by centrifugation at 4000× *g* for 30 s. The samples were then dried with the CentriVap vacuum concentrator and stored at −20 °C until further processing.

### 2.4. LC–MS Analysis

The LC–MS/MS was carried out using a nano-LC coupled to Q Exactive HF Hybrid Quadrupole-Orbitrap Mass Spectrometer (Thermo Scientific, Waltham, MA, USA). Peptides were separated by reverse-phase chromatography using an EASY nLC 1200 system (Thermo Scientific). Peptides were injected into a pre-column (Acclaim PepMap 100, 75 µm × 2 cm, Thermo Scientific), and peptide separation was performed using an EASY-Spray C18 reversed-phase nano-LC column (PepMap RSLC C18, 2 µm, 100A 75 µm × 25 cm, Thermo Scientific) by a gradient of 0.1% formic acid in water (A) and 0.1% formic acid in 80% acetonitrile (B) as follows: from 6% B to 40% B in 80 min; from 40% B to 100% B in 20 min at a flow rate of 0.3 µL/min. Separated peptides were electrosprayed and analyzed using a Q-Exactive HF mass spectrometer (Thermo), operated in positive polarity in a data-dependent mode. Full scans were performed at 120,000 resolutions at a range of 380–1400 *m*/*z*. The top 15 most intense multiple charged ions were isolated (1.2 *m*/*z* isolation window) and fragmented at a resolution of 30,000 with a dynamic exclusion of 30.0 s.

### 2.5. Protein Identification and Quantification

All MS/MS samples were analyzed using Sequest (Thermo Fisher Scientific, San Jose, CA, USA; version IseNode in Proteome Discoverer 2.4.0.305). Sequest was set up to search *S. maximus* and *M. galloprovincialis* proteins against the *S. maximus* genome (49,819 entries, downloaded from National Center for Biotechnology Information database accessed in 24 July 2020) or a custom database integrating *M. galloprovincialis* transcriptome (46,791 sequences) and sequences from the taxa Mollusca retrieved from Universal Protein knowledgebase (UniProt) (335,844 sequences accessed 24 July 2020) [33]. Sequest was searched with a fragment ion mass tolerance of 0.020 Da and a parent ion tolerance of 10 PPM. Carbamidomethyl of cysteine was specified in Sequest as a fixed modification. Oxidation of methionine was specified in Sequest as a variable modification). Scaffold (version Scaffold_4.11.1, Proteome Software Inc., Portland, Oregon) was used to validate MS/MS-based peptide and protein identifications. Peptides were accepted if established at greater than 95.0% probability by the Scaffold local false discovery rate (FDR) algorithm, and proteins were accepted if established at greater than 99.9% probability and contained at least two unique identified peptides. Protein probabilities were assigned by the Protein Prophet algorithm [34]. Proteins that contained similar peptides and could not be differentiated on the basis of MS/MS analysis alone were grouped to satisfy the principles of parsimony. Only proteins sharing significant peptide evidence were grouped into clusters and used for analysis. MS and MS/MS tolerances were set to 10 ppm and 0.6 Da. Trypsin was selected for protein cleavage allowing for one missed cleavage.

### 2.6. Functional Classification of Proteins and Pathway Analysis

Protein functional classification (Gene Ontology, GO) was carried out using the g:Profiler web server version e102_eg49_p15_7a9b4d6 with the database updated on 15 September 2020 [35,36]. Information from this web tool includes pathways from the Kyoto Encyclopedia of Genes and Genomes (KEGG) and REACTOME Pathway database. Protein sequences from *S. maximus* and *M. galloprovincialis* samples were matched to *Danio rerio* (genome assembly GRCz11, NCBI) and *Crassostrea gigas* sequences (genome assembly cgigas_uk_roslin_v1, NCBI), respectively, by running the local BLASTp function from Blast2Go program version 5 (basic), setting a cutoff e-value of 1 × 10^−3^. This analysis was necessary given the lack of annotated genomic information from turbot and Mediterranean mussel. Functional analysis was then carried out on the basis of information from homologous proteins from *D. rerio* and *C. gigas* genomes. Protein sequences were grouped into functional classes (GO classes), employing the g:GOSt function in g:Profiler. A multiquery analysis was carried out for each set of proteins identified from FASP, S-TRAP, and SP3 samples. The *D. rerio* and *C. gigas* genome and the Ensembl database were used as a data source. A hypergeometric test was employed to detect statistically significantly enriched biological processes and pathways in the input protein lists. Results were validated by setting a *p*-value < 0.05 and performing multiple testing corrections using the set counts and sizes (g:SCS) correction method [35].

### 2.7. Protein Quantification

Proteins identified in at least one of the three replicates were considered representative of that sample group. Qualitative information was analyzed with Venn diagrams on Scaffold version 4.11.1 (Proteome Software, Inc, Portland, OR, USA). Protein quantitative data (average precursors ion intensity) were used to report quantitative differences between methods and displayed using R version 4.0.3 (The R Core Team, https://cran.r-project.org/, accessed on October 2020) [37,38].

## 3. Results

### 3.1. Turbot, Scophthalmus Maximus

#### 3.1.1. Peptide and Protein Identification

**Figure 1:**
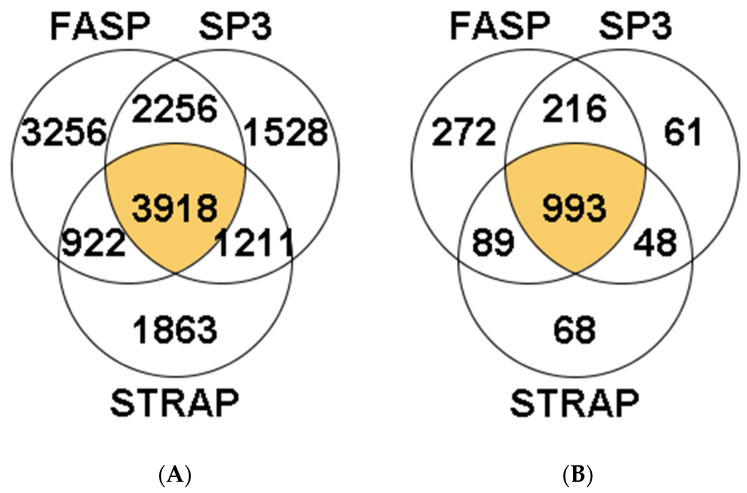
Venn diagrams reporting the total number of shared and unique peptides (**A**) and proteins (**B**) identified using the FASP, S-TRAP, and SP3 methods in turbot liver. Detailed data concerning protein identification are reported in Supplementary Table S1.

A total of 14,954 peptides were identified in *S. maximus* liver samples. Of these, 3918 peptides were identified in the three methods (26.20%). The highest number of peptides was obtained using the FASP method (3256 unique peptides, comprising 21.77% of total identifications, Figure 1A). The percentage of identifications, from the total MS/MS spectra acquired, was approximately 10% in most samples, regardless of the sample preparation method used, except one sample analyzed with the FASP method in which more than 20% of total MS/MS spectra were identified (Supplementary Table S1). Overall, the peptides allowed the identification of 1747 proteins in turbot liver, with 993 of them (56.8%) present in samples from the three methods studied (Figure 1B). Within the remaining 754 proteins, the majority were identified using the FASP method (272 proteins, 36.1% of total identifications), while <10% were identified exclusively using the S-Trap or SP3 methods (68 and 61 proteins, respectively).

**Figure 2:**
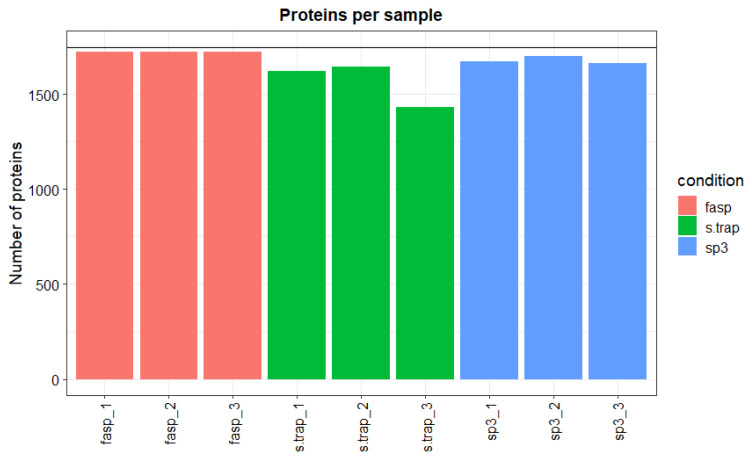
Number of identified proteins per sample in turbot liver.

With regard to the reproducibility of the three methods, we verified that the most reproducible method was FASP with small differences in the total number of proteins identified between sample replicates (total number of proteins varied between 1725 and 1733 in the three sample replicates). The SP3 method was also very consistent among sample replicates retrieving between 1661 and 1702 protein identifications. The S-Trap method was less reproducible, with the number of identifications varying between 1432 and 1647 among sample replicates (Figure 2).

#### 3.1.2. Functional Analysis

To gain insights into the molecular processes in which the identified proteins are involved or play a functional role, we carried out a gene set enrichment analysis employing the bioinformatics tool g:Profiler. A large number of GOs were retrieved by this analysis, enabling a good characterization of the molecular functions and biological processes of all proteins identified in the three sample groups (FASP, S-TRAP, and SP3). In total, nearly 300 GOs were ascribed to FASP, S-Trap, and SP3 proteins (Table 1).

A large number of functions and processes overlapped across the three sets of proteins (FASP, S-TRAP, and SP3), with 79 GO terms related to molecular functions and 222 GOs related to biological processes described for the three sets of proteins.

**Figure 3:**
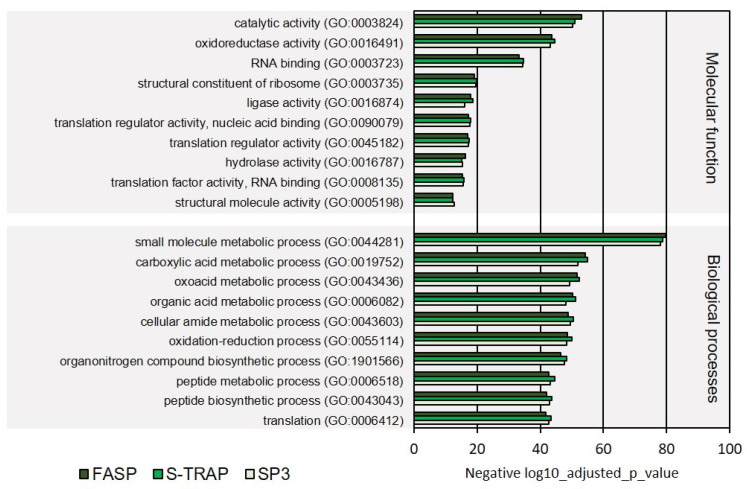
Ten most represented GO terms for molecular function and biological processes on turbot for the three sample preparation methods tested.

**Figure 4:**
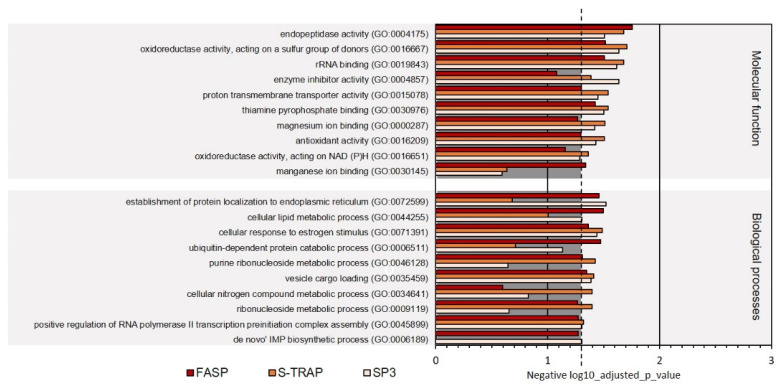
Ten less represented GO terms for molecular function and biological processes on turbot for the three sample preparation methods tested. Dashed line denotes the *p*-value threshold (*p* < 0.05).

In the category molecular functions, proteins with oxidoreductase activity, RNA binding, ligase activity, and translation regulator activity were among the most represented in the three sample groups among ribosomal proteins, proteins with translation regulator activity, hydrolase activity, and other functions (Figure 3, Supplementary Table S2). Proteins classified with endopeptidase activity, oxidoreductase activity, acting on a sulfur group of donors, rRNA binding, enzyme inhibitor activity, proton transmembrane transporter activity, thiamine pyrophosphate binding, magnesium ion binding, antioxidant activity, oxidoreductase activity, acting on NAD(P)H, and manganese ion binding were relatively less abundant in the proteomics datasets retrieved using the three sample preparation methods. The molecular functions differentially represented in the three sample preparation methods were related to magnesium ion binding, antioxidant activity, oxidoreductase activity, and NAD(P)H and manganese ion binding (Figure 4, Supplementary Table S2).

Regarding the GO category biological processes, the majority of the identified proteins were linked to the metabolism of small molecules, carboxylic acids, oxoacids, organic acids, cellular amide, peptide metabolism, oxidation–reduction processes, organonitrogen compound biosynthesis, and translation (Figure 3, detailed list of GOs is displayed in Supplementary Table S2). In the GO classes with lower enrichment, we found proteins with functions related to response to estrogen stimulus, vesicle cargo loading, ribosomal small subunit assembly, one-carbon metabolic process, and posttranscriptional regulation of gene expression (Figure 4, Supplementary Table S2).

Other biological processes were differentially represented in the three sample preparation methods. For instance, more proteins related to the de novo IMP biosynthetic process were identified in SP3 samples, whereas more proteins related to the cellular nitrogen compound metabolic process and ribonucleoside metabolic process were identified in S-Trap, and proteins related to the ubiquitin-dependent protein catabolic process were enriched in FASP (Figure 4, Supplementary Table S2).

**Figure 5:**
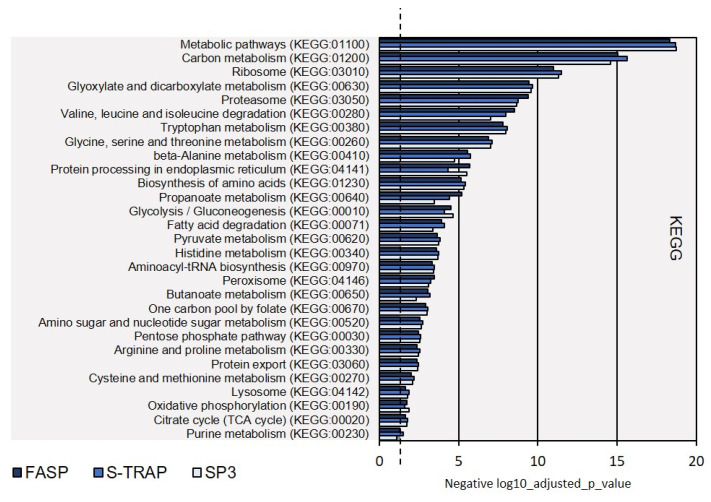
KEGG biological pathways assigned for the proteins extracted on turbot for the three sample preparation methods tested. Dashed line denotes the *p*-value threshold (*p* < 0.05).

We also carried out KEGG (Kyoto Encyclopedia of Genes and Genomes) analysis, as this is complementary to GO analysis to characterize the molecular functions and molecular pathways from the proteomics results. Twenty-nine KEGG pathways were identified from the proteomics results from the three sample preparation methods. The most representative pathways referred to carbon metabolism, ribosome, glyoxylate and dicarboxylate metabolism, proteasome functions, amino-acid metabolism, and protein processing in the endoplasmic reticulum (Figure 5, Supplementary Table S2). Only purine metabolism was not sufficiently represented in the proteomics results retrieved by the S-Trap method (Fisher exact test not significant, *p*-value > 0.05).

### 3.2. Mediterranean Mussel, Mytilus Galloprovincialis

#### 3.2.1. Peptide and Protein Identification

**Figure 6:**
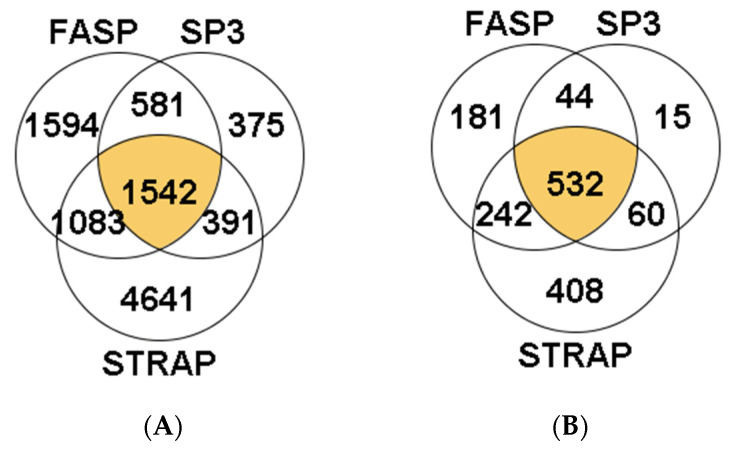
Venn diagrams reporting the total number of shared and unique peptides (**A**) and proteins (**B**) identified using the FASP, S-TRAP, and SP3 methods in the hepatopancreas of *M. galloprovincialis*. Detailed data concerning protein identification are reported in Supplementary Table S3.

A total of 10,207 peptides were identified in the hepatopancreas of *M. galloprovincialis*. Nearly 15% of all these peptides were identified in the three sample preparation methods (1542 peptides). The S-TRAP method enabled the identification of more peptides (4641 unique peptides, 45.5% of total identifications, Figure 6A). Concerning the total MM/MS spectra acquired using the S-Trap method, identified peptides represented between 13.72% and 18.62% of these spectra (Supplementary Table S3). The percentage of identifications was, however, much lower in the case of SP3 (peptides representing between 0% and 6.42% of the MS/MS spectra acquired by the instrument, Supplementary Table S3). Overall, the information collected at the peptide level allowed the identification of a total of 1482 proteins, with 532 of them simultaneously in replicates from the three sample preparation methods (35.9% of the total of proteins identified, Figure 6B). Within the remaining 950 proteins, most were identified exclusively in the S-Trap method (408 proteins, 25.5% of the total number of proteins identified), followed by 181 exclusive proteins identified using FASP (12.2% of the total number of proteins) and only 15 exclusive proteins identified in SP3 method (1% of the total number of proteins).

**Figure 7:**
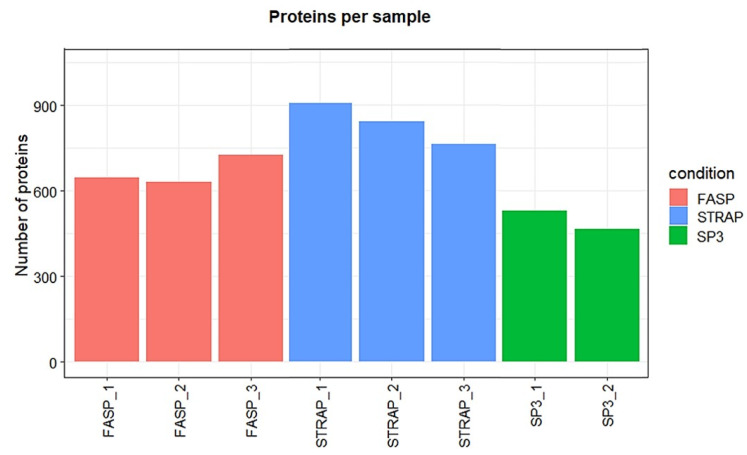
Number of identified proteins per sample in *M. galloprovincialis* hepatopancreas.

Moreover, the S-Trap method allowed the identification of the highest number of proteins per replicate (between 763 and 907), followed by FASP (between 632 and 727, Figure 7). The lowest number of proteins in each replicate was obtained with SP3. Clearly, there was a shortcoming with one of the SP3 replicate samples, in which only seven proteins were identified. Due to this result, one SP3 sample replicate was discarded from the analysis. A total of 467 and 529 proteins were identified in the two remaining SP3 replicates.

#### 3.2.2. Functional Analysis

The functional analysis in g:Profiler revealed 107 GO terms in mussel hepatopancreas proteins identified by SP3 and nearly 150 proteins identified using the FASP and S-Trap methods (Table 2 and Supplementary Table S4). This can be considered a good number of GOs, considering the reduced functional annotation of *M. galloprovincialis* genes [39].

The overall number of GO terms related to molecular function was very similar between FASP and S-Trap samples (63 and 58, respectively, with a total of 45 GO terms overlapping for the two methods). The GO terms retrieved using the SP3 method were lower for molecular function (32) when compared to the other two methods and, therefore, SP3 was unable to provide a good description of several molecular and biological processes.

**Figure 8:**
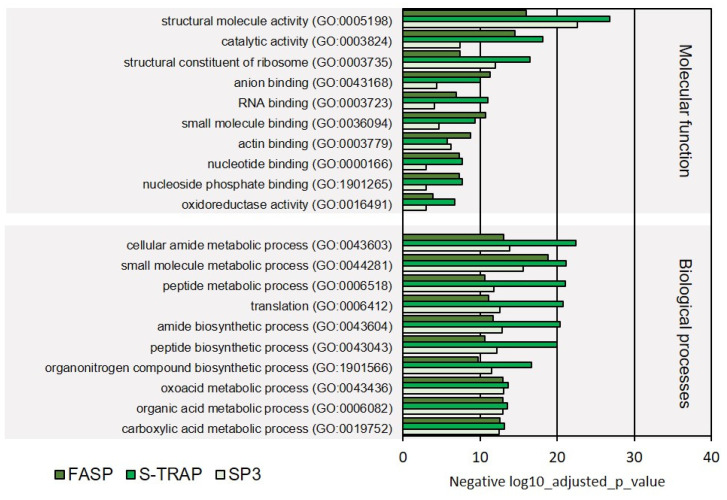
Ten most represented GO terms for molecular function and biological processes on Mediterranean mussel for the three sample preparation methods tested.

The molecular functions most represented in the proteins identified were related to molecule activity, catalytic activity, structural constituent of ribosome, anion binding, RNA binding, small molecule binding, and other intracellular molecular functions. These functional classes were all significantly represented in the proteomics results retrieved by the three sample preparation methods (Figure 8, Supplementary Table S4), with most of these GOs also being reported in the proteomic results from turbot liver.

**Figure 9:**
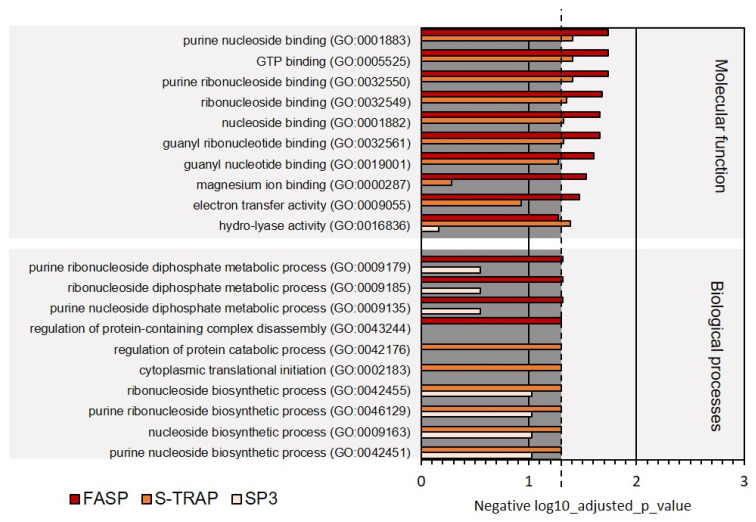
Ten less represented GO terms for molecular function and biological processes on Mediterranean mussel for the three sample preparation methods tested. Dashed line denotes the *p*-value threshold (*p* < 0.05).

More specialized GO terms were also revealed in the functional analysis; however, fewer proteins were associated with these terms, leading to a lower representation of these functions. Among these GO terms, we highlight the molecular functions related to purine nucleoside binding, GTP binding, magnesium ion binding, electron transfer activity, hydro-lyase activity. These functions, unlike the others holding more proteins, were not significantly represented in all three proteomic datasets retrieved by the three sample preparation methods (Figure 9, Supplementary Table S4).

Regarding the biological processes, once again, primary metabolic processes, related to the metabolism of amides, small molecules, peptides, organonitrogen compounds, oxoacids, organic acids, and carboxylic acids, were the most represented comprising the majority of the proteins identified by the three sampling preparation methods (Figure 8). On the other hand, several biological processes related to molecular regulation (regulation of protein-containing complex disassembly, regulation of protein catabolism), translation initiation, and biosynthesis of ribonucleosides were relatively less represented (comprised fewer proteins) in this functional analysis (Figure 9). Several of these functions were not significantly represented in all three proteomic datasets retrieved by the three sample preparation methods.

**Figure 10:**
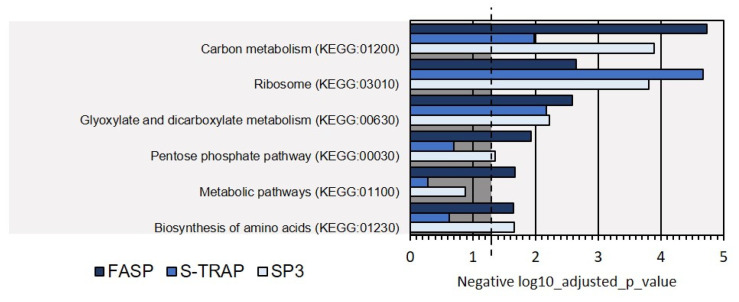
KEGG biological pathways assigned for the proteins extracted on Mediterranean mussel for the three sample preparation methods tested. Dashed line denotes the *p*-value threshold (*p* < 0.05).

Moreover, the KEGG analysis identified some of the biological pathways represented at the protein level in the proteomics analysis with the three sample preparation methods. This analysis revealed that most of the proteins identified by the three sample preparation methods were related to carbon metabolism and ribosome functions. Other pathways comprised pentose phosphate and amino-acid synthesis (Figure 10). However, not all pathways were equally represented at the protein level, as reflected in the *p*-value of the Fisher exact test, and some pathways (e.g., biosynthesis of amino acids and pentose phosphate) were not evident in the proteomics data retrieved using the S-Trap method, for instance (*p* > 0.05).

### 3.3. Protein Markers of Oxidative Stress and Response to Toxic Substances

**Figure 11:**
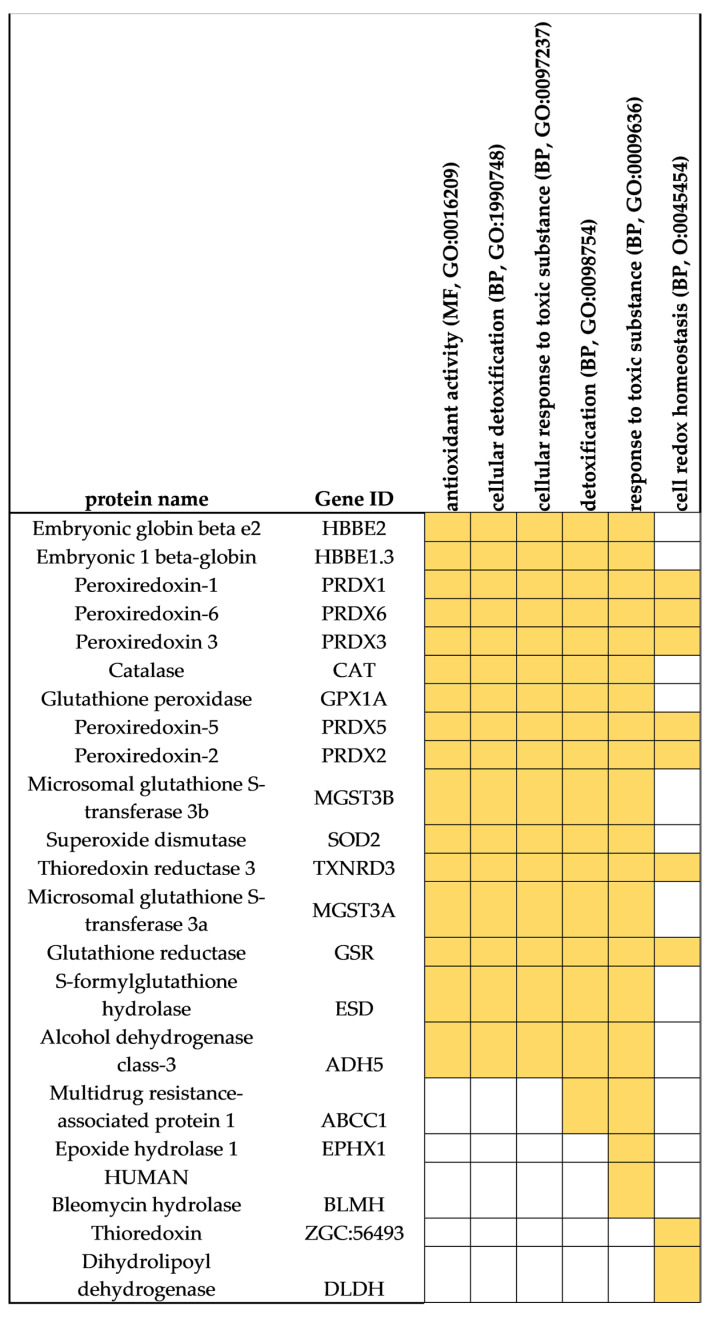
Proteins from *S. maximus* with functions related to antioxidant processes (GO: GO:0016209), cellular response to toxic substance (BP, GO:0097237, GO:0009636), detoxification (BP, GO:1990748, GO:0098754), and cell redox homeostasis (BP, GO:0045454).

We then aimed to explore in more detail some protein functions by searching for individual sets of proteins identified by the three sample preparation methods. The focus of this analysis was protein markers involved in the oxidative stress and response to toxic substances (detoxification), as these are considered of relevance to understand the physiology of these species and their responses to environmental challenges in aquaculture. In *S. maximus* liver, several functional categories related to these processes were found, namely, antioxidant activity (GO:0016209), cellular detoxification (BP, GO:1990748), cellular response to toxic substance (BP, GO:0097237), detoxification (BP, GO:0098754), response to toxic substance (BP, GO:0009636), and cell redox homeostasis (BP, GO:0045454). Therefore, we examined all the proteins included in these functional categories. The analysis revealed, for instance, a significant number of enzymes from the antioxidant system of this species (Figure 11). Among the antioxidant enzymes identified were peroxiredoxins (five isoforms), catalase, glutathione peroxidase, glutathione reductase, thioredoxin reductase, thioredoxin, and embryonic globin beta that participate in hydrogen peroxide elimination mechanisms. Other key enzymes of the antioxidant system identified were superoxide dismutase, glutathione *S*-transferase (two isoforms), *S*-formylglutathione hydrolase, and alcohol dehydrogenase. This analysis suggests that all methods may be used for carrying systematic studies targeting the antioxidant system in fish [40,41]. Other proteins identified using these methods are also highlighted here, given their importance in processes related to exposure to chemical substances and contamination. In addition to GSTs, which represent key enzymes from phase II xenobiotic metabolism, other enzymes identified in turbot liver included multidrug resistance-associated protein 1, known to be responsible for the transport and elimination of several toxic compounds (phase III mechanisms from xenobiotic metabolism), epoxide hydrolase 1, and human bleomycin, involved in the biotransformation of toxic compounds [8,42].

Unfortunately, in Mediterranean mussel, no functional categories related to oxidative stress and response to toxic substances were found. The absence of these functional categories may imply a lack of identification of proteins related to these functions. However, the low level of annotation of many *M. galloprovincialis* proteins can also place difficulties in the functional analysis and in the proper identification of the abovementioned functional categories [11,33]. Indeed, a subsequent search in the list of total proteins identified in *M. galloprovincialis* (Supplementary Table S3) revealed that most of the protein markers found in *S. maximus* and listed in Figure 11 were also identified in *M. galloprovincialis* [43]. Among these protein markers were peroxiredoxins, glutathione peroxidase, glutathione *S*-transferase, superoxide dismutase, thioredoxin reductase, thioredoxin, thioredoxin peroxidase, glutathione reductase, *S*-formylglutathione hydrolase, alcohol dehydrogenase class-3, multidrug resistance-associated protein 1, epoxide hydrolase, and dihydrolipoyl dehydrogenase.

## 4. Discussion

Regarding the total number of proteins identified, in general, the three sample preparation methods showed similar performances for the same type of tissue. However, the performances were distinct when comparing the results between tissues and species studied. The results showed a decrease in the number of proteins identified in the hepatopancreas of Mediterranean mussel, compared to the liver of turbot. This lower protein identification applied to all sample preparation methods studied. This result was expected, to some extent, given the high genetic variability that characterizes this species. In fact, the genome of this species presents a very complex architecture, presenting an extremely high number of “dispensable” genes whose presence and absence vary from individual to individual [44]. Much of this genomic information is not included in the reference genome of this species and is, therefore, substantially uncharacterized [44].

FASP demonstrated good performances in both types of tissues, consistent with the great versatility and wide use of this method in proteomics studies. SP3 showed good performance in turbot liver samples (highest number of protein identifications following FASP), but low performance in Mediterranean mussel hepatopancreas samples. A note should be given to the fact that different digestion enzymes were suggested by the authors of this method [21]. The use of the mix with trypsin and rLysine-C is known to enhance proteolysis and provide multiple positive effects on protein mass spectrometry analysis. This procedure, however, did not improve the performance of SP3 over FASP. S-Trap showed distinct performances in both tissues, producing the lowest number of identifications in turbot liver and the highest number of identifications in the Mediterranean mussel hepatopancreas.

Previous work has shown that the SP3 method performances are similar or even superior to FASP and other proteomic sample preparation methods. The performance of the SP3 method has been shown to be superior, allowing a greater number of peptide and protein identifications, especially from samples with low amounts of protein (<10 µg total protein) [45,46]. The high performance of SP3 has been attributed to the independence of this method from protein molecular weight. For example, in the FASP method, the use of centrifugal filter units for buffer exchange and elimination of contaminants from the samples leads to the partial loss of samples (protein fractions below 10 or 30 kDa) [47]. A comparative study of the performance of the three methods (FASP, S-TRAP, and SP3), with tissue lysates from the plant *Arabidopsis thaliana*, revealed total peptide recovery percentages (after digestion) slightly higher with SP3 relative to FASP and a significantly low peptide yield with the S-Trap method. In the same study, SP3 and FASP revealed a greater number of peptide and protein identifications and lower coefficients of variation in protein quantitation compared to the S-Trap method [46]. The superior performance of the method SP3 was also verified in another study that addressed proteomes from bacteria [48]. Notably, the type of biological material can greatly determine the performance of each sample preparation method. For example, in the proteome analysis of the spore proteome of the fungus obligate biotroph *Spongospora subterranea*, the S-Trap method proved to be more efficient than the SP3 method, allowing the identification of an increased number of proteins from this biological material [49].

However, regarding the SP3 method, it is important to note that the binding process of proteins to paramagnetic beads proved to be quite sensitive to pH. The performance of this method depends on the efficiency of this binding. A very acidic medium reduces protein binding, leading to subsequent sample loss and low performance (reduced peptide recovery, as well as peptide and protein identification). This condition may explain, in part, the poor performance of the method in proteomic samples from Mediterranean mussel. Indeed, despite the use of a high amount of total protein (100 µg) we verified a relatively low peptide yield, suggesting significant sample loss with this method. With regard to this issue, it will be important, in future applications of the SP3 method, to monitor the pH of the sample and the process of protein binding to the paramagnetic beads, as suggested by Sielaff [45]. The acid hydrolysis of DNA and RNA, during the preparation of protein extract, proved to be compatible with SP3, enhancing the efficiency and performance of the method, as well as reducing the interference of these molecules in the analysis of crude protein extracts [45].

Despite its versatility and robustness, one of the biggest bottlenecks of the FASP method is the significant loss of sample, often related to the use of centrifugal filter units (a requisite of this method). This loss was shown to be particularly critical in the case of biological samples with a low total amount of protein. Under these conditions, FASP reveals low performance, leading to a low number of identifications and high quantitative variability [45]. Some variations of the FASP method have been proposed in order to improve its performance. Among the suggested modifications is the use of polyvinylpyrrolidone. This compound significantly improves the yield of the method, and its application in samples with a low amount of protein (<10 μg) prevents precipitation and agglomeration and increases the solubility of proteins in solution [50]. Fractionation strategies of peptide mixtures, using gas chromatography or high-pH reversed-phase chromatography, have also been shown to contribute to improve the overall performance of this method [51]. A miniaturized FASP protocol (micro-FASP) was proposed for processing proteomic samples in the sub-microgram range (∼0.01–0.2 μg total protein). This method relies on the use of ultrafiltration devices with filters with ∼0.1 mm^2^ surface area to reduce the total volume of reagents and samples to less than 10 μL [52].

Other sample preparation methods have been proposed and tested as alternatives to FASP, S-TRAP, and SP3. Alternative methods include in-stage-tip (iST), in-solution digestion (ISD), gel-aided sample preparation (GASP), or IP-SP3, which is a method specifically dedicated to protein interactome studies [45,51,53]. Some of these methods can be used together with FASP, S-TRAP, or SP3 methods, to complement the proteomics studies and enhance proteome coverage [51].

## 5. Conclusions

Although some omics studies in aquaculture species are becoming more common, not many studies dedicated to the optimization of proteomics methodologies using species such as *Mytilus* sp. or *Scophthalmus* sp. are available [54]. The outcome of MS-based proteomic analysis to a great extent depends on the choice of appropriate sample preparation method. Sample preparation for shotgun proteomics consists of several steps, including the extraction and solubilization of proteins, protein denaturation, enzymatic digestion, and the purification of peptides. In the present work, we compared three methodologies (FASP, S-TRAP, and SP3) for processing protein material from two aquaculture species, liver tissue from the fish *S. maximus* and hepatopancreas from the mussel *M. galloprovincialis*.

Overall, a satisfactory proteome coverage was achieved using all three methodologies, enabling protein identification comparable to other proteomic studies of these species and allowing potential characterization of several primary metabolic processes. Taken together, from both species and tissues, FASP was the most efficient method, enabling the highest and second-highest number of protein identifications. On the other hand, FASP was the most time-consuming method, resulting in a lower number of samples being processed per day, in addition to several hands-on steps, potentially increasing the risks of technical errors.

It is clear to state that the GO terms associated with proteins identified using all three methods in Mediterranean mussel were mainly related to primary metabolic processes. When comparing the representation of specific GO terms by each method, it should be mentioned that (ribo)nucleoside biosynthetic process-related GO terms were again not represented in samples from SP3 methods and, to some degree, FASP.

## Figures and Tables

**Table 1:** Total number of GO terms retrieved by g:Profiler in *Scophthalmus maximus*. Detailed information of GO terms identified is reported in Supplementary Table S2.

GO Categories	FASP	S-TRAP	SP3
Molecular Function	76	78	77
Biological Processes	219	215	213
Total	295	293	290

**Table 2:** Total number of GO terms retrieved by g:Profiler in *M. galloprovincialis*. Detailed information of GO terms identified is reported in Supplementary Table S4.

GO Categories	FASP	S-TRAP	SP3
Molecular Function	63	58	32
Biological Processes	95	90	75
Total	158	148	107

## Supplementary Materials

The following are available online at https://www.mdpi.com/article/10.3390/proteomes9040046/s1: Supplementary Table S1: Peptides and proteins identifications in *S. maximus*; Supplementary Table S2: gProfiler results for *S. maximus*; Supplementary Table S3: Peptide and protein identifications in *M. galloprovincialis* using distinct sample preparation methods; Supplementary Table S4: gProfiler results for *M. galloprovincialis*.
